# Aging and Modified Washing Process for Polyester Fabrics—Environmental Impact

**DOI:** 10.3390/polym16233238

**Published:** 2024-11-22

**Authors:** Ana Šaravanja, Tanja Pušić, Julija Volmajer Valh, Tihana Dekanić

**Affiliations:** 1 Faculty of Textile Technology, University of Zagreb, HR-10000 Zagreb, Croatia; ana.saravanja@ttf.unizg.hr (A.Š.); tanja.pusic@ttf.unizg.hr (T.P.); 2 Faculty of Mechanical Engineering, University of Maribor, SI-2000 Maribor, Slovenia; julija.volmajer@um.si

**Keywords:** polyester fabric, artificial aging, washing process, wastewater, defragmentation

## Abstract

Aging and washing factors have a direct influence on changing the properties of textile products, e.g., causing a release of textile fragments in the washing process. In this study, polyester fabrics were exposed to artificial aging under controlled conditions. Using a modified washing process, polyester fabrics were subjected to 10 washing cycles before and after the aging process. To monitor the influence of aging and the modified washing process on the polyester fabrics, the physical, structural and morphological properties of the fabrics and the composition of the collected wastewater were analyzed. The results indicate a slight degradation and increased defragmentation of the polyester fabric due to the processes used. Aging caused the phenomenon of “annealing”, photo-oxidative degradation, and the local thickening of the individual fibers. Aging and washing processes influence the change in tensile strength properties. An analysis of zeta potential and BET results confirmed that the aging process results in surface modifications that depend on the time of exposure. The physico-chemical characterization and microscopic analysis of the wastewater revealed various fragments and short, detached fibrils. The results confirmed that both aging and washing significantly affect the properties of polyester fabrics and the composition of the wastewater resulting from the washing process. The relevance of this research to environmental matters is emphasized through the parameters chosen, which reveal the influence of aging on polyester fabric characteristics and the contamination detected in wash wastewater. In conclusion, several avenues for future research have been identified, including lowering washing temperatures, choosing more appropriate detergents, and adjusting standard washing protocols.

## 1. Introduction

Polyester fibers are synthetic polymers that originate from petroleum-derived chemicals. Chemically, these fibers are primarily made up of long-chain polymers characterized by a backbone containing functional ester groups (-COO-). The most common type of polyester used in textiles is polyethylene terephthalate (PET) [1,2,3], which is gaining popularity for its excellent performance and structural characteristics, including strength, durability, and resistance to creasing, shrinkage, and abrasion. [4,5]. In the last two decades, production has seen a twofold increase, and forecasts suggest it will reach 70 million tons by 2030 [6]. Polyester fibers are hydrophobic, demonstrating the minimal absorbency of water and moisture from the atmosphere, resulting in their inability to retain these substances. These qualities allow polyester textiles to dry swiftly [7] and make them simple to care for and maintain. They are widely used and are often combined with other fibers such as cotton and wool to improve certain properties, e.g., mechanical strength, dimensional stability, thermal stability, and comfort [6,7]. Negative performance characteristics include a tendency to flake, pilling, stiffness, static electricity charging, and hydrophobicity [5,8,9]. The environmental impact of polyester fibers includes their dependence on non-renewable resources for production and their limited biodegradability [3,9,10].

**Figure 1:**
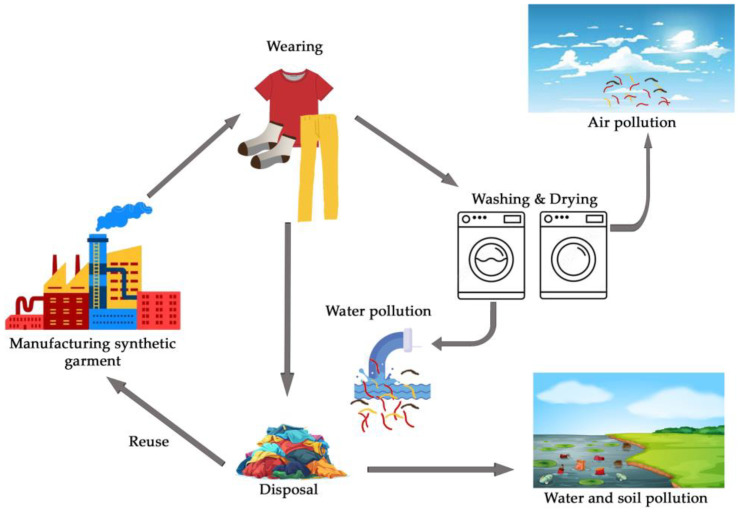
Life cycle of textile products and their impact on the environment.

The inclination of textile materials to emit fiber fragments, especially those that are synthetic, including polyester, polyamide, and polyurethane, has become a critical environmental concern. The release of fiber fragments occurs at different stages of the life cycle of textile products, including manufacturing, wearing, washing, and disposal (Figure 1). The main factors contributing to this trend are primarily the washing process and garment care [11,12,13,14,15,16,17,18]. Due to the fabric structure, which consists of staple fibers or long, continuous filaments, the polyester surface can be broken or abraded under various mechanical impacts, resulting in the release of smaller fragments [19,20]. Once released and detached from textiles, microfiber fragments can accumulate in water bodies over days and years, potentially harming marine life and entering the food chain [17,19,20,21]. Efforts to mitigate the release of polyester fiber fragments include the development of technologies to capture microfiber fragments during the washing process, filtration systems promoting the use of laundry bags or filters, advocacy for the use of more sustainable and less shedding textile materials, and improved fabric and garment design [14,20,22,23,24,25,26]. Most tests are performed according to standards using a specific detergent, while some standards involve washing in water.

In 2018, five industry associations, including AISE, CIRFS, EOG, EURATEX, and FES, came together to proactively address the environmental issue of microplastics. One step towards a solution was the exchange of knowledge and information. As a result, a test method was developed which, once evaluated, will become an official CEN standard [27]. In 2021, the American Association of Textile Chemists and Colorists (AATCC) developed and introduced the first test method for the release of fiber fragments during home laundering (AATCC TM212-2021, Test Method for Fiber Fragment Release During Home Laundering). Textiles are known to release “microfibres” into the environment, and these tiny particles are recognized as pollutants due to their small dimensions. The term “microfiber’ is synonymous with “fiber fragment” in the context of the release of fibers during household washing, with the AATCC stating that ‘microfiber’ is only associated as a linear density category [28].

In addition to the influence of the washing process, there are several other processes that affect the properties of textiles. The aging of the material is one of them. Natural aging and artificial aging are two different processes that simulate the effects of time and environmental influences on materials. Both processes provide valuable insights into the long-term durability and performance of materials under real-life conditions. Natural aging occurs over time when materials are exposed to environmental influences. It refers to changes in polymer materials that occur over a period of time in the natural environment [29,30]. In order to obtain accurate data for analysis, such materials must be exposed to natural atmospheric conditions over an extremely long period of time, often months or years. Due to the long duration of the natural aging process, researchers are turning to the concept of artificial aging, which can produce similar effects but offers better control and reproducibility [31].

A significant number of researchers are examining how fiber fragments are released from polyester textiles throughout the washing process. Their studies focus on different variables, including the material’s type and structure [32,33,34,35,36], its functional processing [37,38,39,40,41], and the washing conditions, which encompass the type of detergent used, the water characteristics, the application of softeners, the addition of washing balls, etc., [23,42,43,44,45]. There is a relatively small group of researchers examining the connection between the artificial aging process and the fragmentation of polyester materials that occurs during washing [44,46,47].

The focus of this research is to analyze how varying artificial aging times affect the defragmentation of standard polyester fabric during a modified washing process conducted over ten cycles, as opposed to unwashed fabric. This work contributes to a deeper understanding of the underlying cause-and-effect relationship and offers new insights relative to previously published studies.

Polyester fabrics were subjected to artificial aging simulation under laboratory conditions at different time intervals. The untreated and aged samples were then subjected to the modified washing process over 10 cycles. The washing process consists of two phases—the first is the washing process according to the standard HRN EN ISO 6330 protocol, and the second is rinsing. The process used was a modified rinsing protocol, according to which the rinsing bath was gradually cooled from 60 °C (the first rinsing cycle), 50 °C (the second rinsing cycle), 40 °C (the third) and 30 °C (the last) in order to achieve the slow relaxation and stabilization of the polyester fabrics.

**Figure 2:**
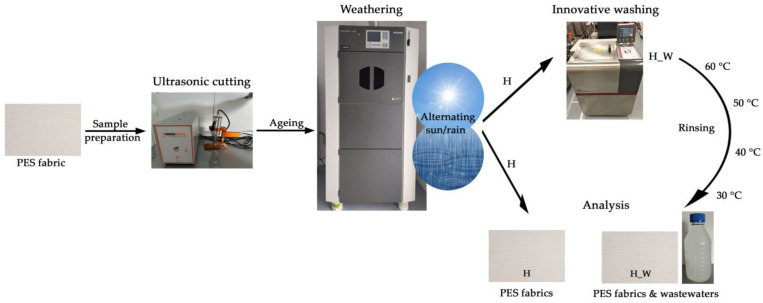
A schematic representation of the workflow.

The study examined the physical, structural, and morphological attributes of untreated and aged fabrics, assessing changes that occurred before and after they were subjected to 10 washes. The wastewater produced from ten washing sessions was collected and subjected to analysis through physico-chemical parameters to investigate the effects of polyester fabric aging on the wastewater composition. Figure 2 presents a schematic depiction of the workflow.

## 2. Materials and Methods

### 2.1. Material

For the purposes of this research, a standard bleached fabric made from 100% polyester in plain weave was employed. This fabric, obtained from the Center for Test Materials (CFT) in Vlaardingen, the Netherlands, features a mass per unit area of 156.0 g/m^2^, a thickness of 0.35 mm, and a thread density of 27.7 warp and 20.0 weft threads per centimeter, along with a warp/weft fineness of 30.4/31.9 tex.

The samples were cut using the TTS400 ultrasonic cutter from Sonowave, Legnano, MI, Italy, to eliminate the potential influence of threads that might protrude from their edges.

### 2.2. Artificial Aging

The fabric was exposed to accelerated artificial aging (H) under controlled conditions in the Xenotest 440, SDL Atlas, Mount Prospect, IL, USA, within 5 cycles of 26 h, including alternating sun (dry) and rain (wet) periods, according to ISO 4892-2+A1:2013 [48]. The exposure cycle included a dry phase lasting 102 min, followed by an 18 min water-spraying phase. The irradiance was set to 60 W/m^2^, with the chamber temperature at 38 °C, the black standard temperature at 65 °C, and relative humidity maintained at 50%, across a wavelength range of 300 to 400 nm.

### 2.3. Washing Procedures

Polyester fabrics were subjected to washing before (N) and after aging (H) in accordance with HRN EN ISO 6330:2012 [49], following procedure 2A. The washing process utilized 1.25 g/L of ECE A standard detergent in tap water, maintaining a bath ratio of 1:7. The fabrics underwent 10 wash cycles at 60 °C for a duration of 30 min each, using the Rotawash machine from SDL Atlas. After washing, the four rinse cycles were performed according to a modified protocol [50]. The rinsing bath temperature was systematically reduced from 60 °C to 50 °C, then to 40 °C, and finally to 30 °C, facilitating a gradual relaxation of the polyester fabrics.

The wastewater generated from the advanced washing cycles was gathered and examined through physico-chemical parameters to assess the fragments and various soluble and insoluble substances released from the polyester fabrics. To avoid the formation and loss of protruding fibrils and fragments during successive wash cycles, the samples were air dried. The samples were not dried between the individual cycles. For this objective, only the polyester fabrics from the 10th wash were air-dried in a flat position.

Table 1 presents the labels and descriptions of polyester fabrics prior to and following the artificial aging and washing processes.

The wastewater gathered from each subsequent modified washing cycle was carefully blended to create a representative sample, which was then subjected to filtration through membrane vacuum filtration utilizing a polyethersulfone membrane filter (Sartorius) with a pore size of 0.2 µm.

### 2.4. Methods

#### 2.4.1. Characterization of Polyester Fabrics

The Kern OBE 134, KERN & SOHN GmbH, Balingen, Germany transmitted light microscope was used to observe changes and differences in the pore size and shape of treated polyester fabrics compared to untreated fabrics.

A Premier-type digital microscope, Dino-Lite AM7013MZT, AnMo Electronics Corporation, New Taipei City, Taiwan was used to analyze the surface of certain yarns of untreated and treated fabrics.

Surface changes in aged and aged/washed samples were analyzed with a Tescan VEGA scanning electron microscope, manufactured in Brno-Kohoutovice, the Czech Republic, at a magnification level of 1000×.

The breaking force (F_b_) and elongation at break (ε_b_) of both untreated and treated fabrics were measured in the warp direction utilizing a Tensolab Strength Tester from Mesdan S.P.A., Raffa, BS, Italy. The testing was conducted with a preload ranging from 5 to 7 N and a gauge length of 200 mm ± 1 mm.

The zeta potential (ZP) of all polyester fabrics was determined in the SurPASS electrokinetic analyzer, A. Paar, Graz, Austria using the streaming potential method in the pH range from 10 to 2.5 of the electrolyte solution, 0.001 mol/L KCl. The ZP was calculated instrumentally using the Helmholtz–Smoluchowski equation [51].

The specific surface area (SSA) of the polyester fabrics was determined using the Brunauer–Emmett–Teller method (BET) on a Gemini 2380 Surface Area Analyzer, Micromeritics, Norcross, GA, USA, by nitrogen adsorption at the temperature of liquid nitrogen (t = −196 °C).

The gravimetric analysis of the polyester fabrics before and after aging and washing was carried out on a laboratory scale.

#### 2.4.2. Washing Wastewater Characterization

A Kern OBE 134 transmitted light microscope was used to examine the collected wastewater samples in order to detect microfiber fragments.

The turbidity of the wastewater was assessed using the turbidimeter TL2350 Hach, Loveland, CO, USA, following the guidelines of HRN EN ISO 7027-1:2016 [52]. This assessment involved taking three separate measurements for each wastewater sample, with the results presented as an average value.

The total suspended solids (TSS) concentration in the wastewater was assessed by performing vacuum membrane filtration on 150 mL of a thoroughly mixed water sample. This was accomplished using a pre-weighed polyethersulfone filter and employing the gravimetric method. After the filtration step, the residue collected on the filter is dried at 105 °C until a constant weight is achieved.

The total solids content (TS) and the total dissolved solids content (TDS) are determined using a weighed porcelain dish. For the TS determination, 100 mL of the wastewater sample is evaporated at 103–105 °C and then completely dried in a dryer; the residue is then cooled in a desiccator and weighed. For the TDS content, the 100 mL filtrate sample is evaporated in the same way as TS.

The pH value and conductivity of the wastewater were determined using a Mettler Toledo multimeter, Greifensee, Switzerland. The determination method is based on the standards HRN EN 27888:2008 and HRN EN ISO 10523:2012 [53,54].

The chemical oxygen demand (COD) is determined by a cuvette test in which 3 mL of wastewater is pipetted into an ampoule containing sulphuric acid, the oxidizing agent K_2_Cr_2_O_7_, and the catalyst Ag_2_SO_4_, and then heated for 2 h at 148 °C in a WTW CR2200 device. The ampoules are then cooled to room temperature and the absorbance and oxygen concentration (mg O_2_/L) are measured with PhotoLab S6, WTW, Weilheim, Germany.

## 3. Results and Discussion

The research focused on examining the impacts of weathering and washing, which were influenced by the properties of the polyester fabrics and the parameters of the wastewater. Special attention was given to the released solid fragments to assess the correlation between the composition of the materials and the wastewater, with both aspects highlighting their environmental implications, including the degradation and fragmentation of polyester fabrics and the resulting pollution of wastewater.

The analysis of the polyester fabrics, namely untreated (PES_N), untreated–washed (PES_N_W), aged (H), and aged–washed fabrics (H_W), included tensile strength properties, a microscopic observation of the fabric and warp yarns, surface charge, and specific surface area. The characterization of the wastewater produced in the washing process was carried out using TDS, TSS, TS, turbidity, pH value, conductivity and microscopic assessment.

### 3.1. An Analysis of the Properties of Polyester Fabrics

Table 2 illustrates standard polyester samples prior to and following artificial aging and washing, observed at a magnification of 40×.

**Figure 3:**
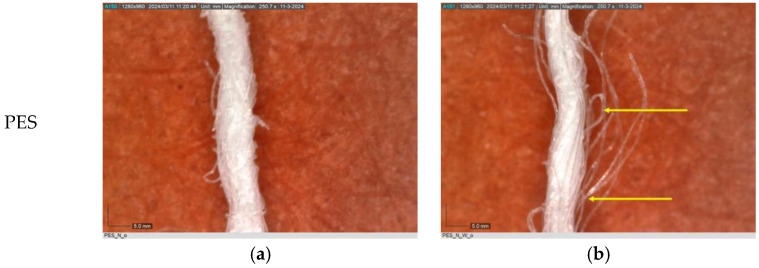
Individual warp yarn of polyester fabrics: (**a**) untreated; (**b**) washed.

The observations of polyester samples presented in Table 2 do not provide sufficient insight for assessing the effects of aging and washing. Therefore, individual warp yarns have been isolated and examined at a higher magnification (250×), as shown in Figure 2 and Figure 3.

In Figure 3, untreated polyester yarn (PES_N) is compared to yarn that has been subjected to 10 wash cycles (PES_N_W). The results indicate that the polyester fabric experiences changes during washing, leading to the fibers protruding (yellow arrow) from the yarn (Figure 3b).

**Figure 4:**
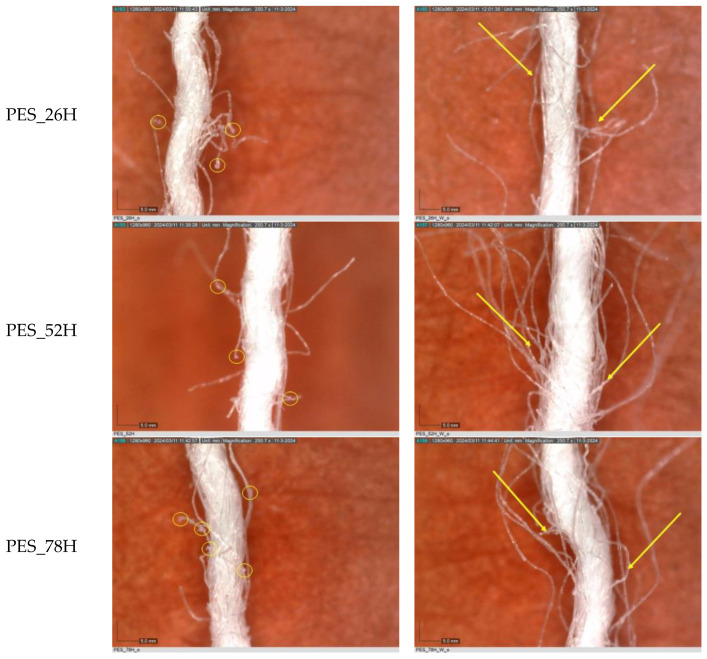

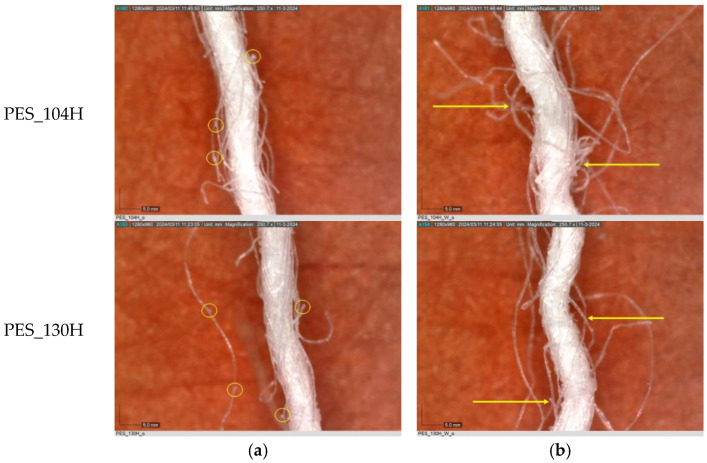
Individual warp yarn polyester fabrics: (**a**) aged; (**b**) aged and washed.

The images captured using a digital microscope at a magnification of 250× provide a clearer view of the surface of the individual warp yarns. Figure 4a shows individual yarn segments of aged polyester samples, and aged and washed samples (Figure 4b) as a function of aging time. With increasing aging time, a slight degradation of the fabric occurs; surface changes due to slight deformation of the yarn were observed (slightly protruding fibers and the local thickening of individual fibers, in particular PES_78H and PES_104H; Figure 4a). The reason for the thickening of the polyester fibers during aging is the phenomenon of “annealing” (yellow circles), in which the molecules in the polymer chains rearrange themselves, leading to changes in the structure and properties of the fiber. The molecular relaxation and rearrangement led to an increase in the thickness or diameter of the polyester fibers [55,56]. The images show a difference in the number and length of fibers protruding from the structure of the polyester fabric. The washing process leads to increased fibrillation, defragmentation, and slight deformation, as well as to a deterioration in the morphological properties of the yarn (Figure 4b). This phenomenon can be attributed to both the mechanical action involved in the washing process and the impact of the detergent on the polyester fabric. Washing polyester fabric in a detergent solution with a slight alkalinity at 60 °C promotes hydrolysis of the fibers, resulting in surface etching. This phenomenon is corroborated by the rise in fibrillation observed (Figure 4b). Additional washing causes the fibril bundles to break apart and appear on the surface of the material [55,56], negatively affecting the overall surface of the fabric.

The tensile properties play a crucial role in assessing structural changes. Consequently, the extent of alterations resulting from artificial aging and washing was carefully observed.

The results displayed in Table 3 illustrate the breaking force (F_b_) and elongation at break (εb) for polyester fabric samples, evaluated before and after undergoing artificial aging and ten washing cycles. The breaking force measurements were conducted in triplicate, with the average value provided.

According to the data in Table 3, the maximum breaking force of the standard polyester fabric is 58.4571 MPa, which can be regarded as a good strength of the polymer structure. The impact of artificial aging on standard polyester fabrics leads to changes in the mechanical properties, which is reflected in a change in the breaking force values. Prolonged exposure diminishes tensile strength, leading to the brittleness of polyester when subjected to further embedding. The tensile strength of polyester fabrics diminished as the duration of weathering cycles increased. These findings are consistent with earlier research. [57].

The washing of standard polyester fabrics for 10 cycles (PES_N_W) resulted in an increase in breaking strength, as evidenced by a breaking force value of 58.6111 MPa. The impact of 10 wash cycles on the tensile properties of aged fabric is marked by a modest rise in breaking force when compared to the aged samples.

A comparison of the aged samples (H) with those that were both aged and washed (H_W) reveals that the washing process positively influences the tensile properties, demonstrated by a 4.1379 MPa increase in maximum force (ΔFmax) from PES_104H to PES_104H_W. Assessing the fabric dimensions both pre and post wash demonstrated that shrinkage was not the factor leading to the increase in breaking force.

**Figure 5:**
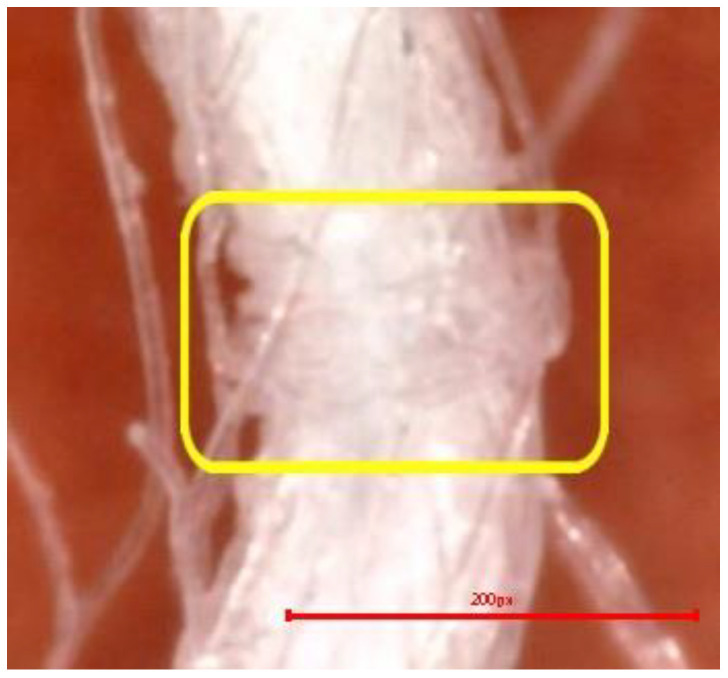
Formation of loops around yarn.

Consequently, a magnified portion of the yarn (Figure 4b) is illustrated in Figure 5, representing a structural component of the aged and washed fabric sample (PES_130H_W).

The enlarged, emphasized segment of the aged washed yarn shows the formation of loops, which proves that the washing parameters have caused changes in the polyester yarn. The protruding fibril fragments within the yarn structure led to the formation of loops around adjacent threads or crossing points within the fabric. The formation of loops around the yarn during the washing process is the result of the synergy of the aging and Sinner parameters of the washing process. Figure 5 shows that the initial fragmentation starts with aging, so this could be a primary effect. The effects of aging can be linked to molecular-level changes, including the oxidation of polymer chains and the breaking of bonds that lead to the formation of new molecules [58,59]. The secondary effect occurs during the wash process due to the mechanical agitation, temperature, and friction in a washing container, whereby the water flow can also contribute to the entanglement of the threads. If the tension across the fabric threads is inconsistent or if certain areas exhibit higher tension, the chances of loops forming may rise as the fabric shifts during washing. The improvement in tensile properties for all washed polyester fabrics, as indicated in Table 3, results from the formation of loops.

**Figure 6:**
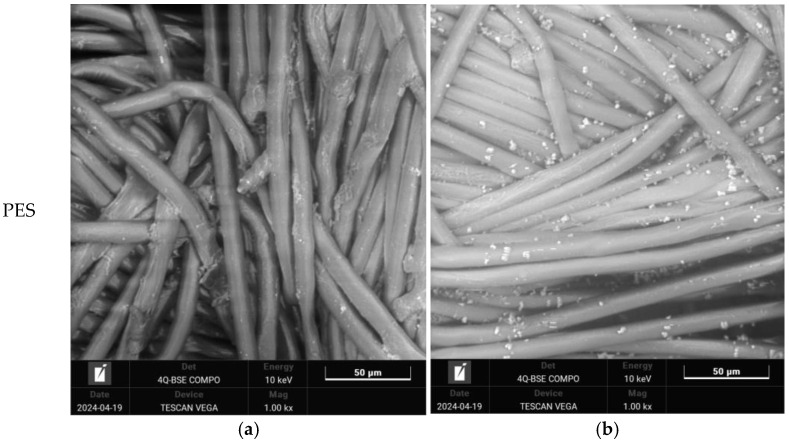
SEM images of polyester fabrics at 1000× magnification: (**a**) untreated (PES_N); (**b**) untreated–washed (PES_N_W).

**Figure 7:**
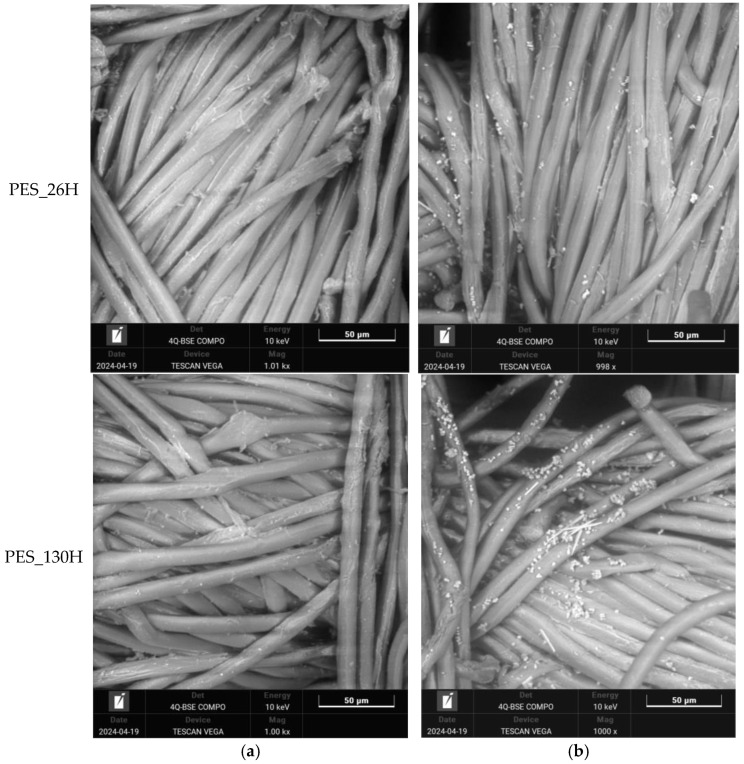
Scanning electron microscope (SEM) images of polyester fabrics at a magnification of 1000×: (**a**) aged sample, (**b**) aged and washed sample.

The impact of both aging and washing on polyester fabrics was analyzed using a scanning electron microscope with a magnification of 1000×.; see Figure 6 and Figure 7. The selection of samples for SEM analysis included untreated, 26 h, and 130 h aged samples and their washed equivalents.

Some solid particles are visible on the untreated–washed polyester fabric (Figure 6b), although no aging treatment was carried out. The reason for this is the detergent and its composition. The SEM images show that aging causes the photooxidative degradation of the polyester surface, which is visible as the destruction of the fiber surface (PES_26H and PES_130H). It is apparent that the washing process affects the surface properties of aged polyester fabric. The SEM images illustrate modifications in the washed samples, with a notable buildup of calcite particles on the surface, especially prominent in the sample that has been aged for 130 h. Over an extended period of aging, the interplay of sun and rain cycles led to the erosion of the polyester surface, as the water removed the portions that had degraded due to sunlight exposure. An eroded surface has a greater tendency to attract and hold onto deposits and calcite particles compared to untreated samples and those aged for 26 h. Consequently, an increased exposure time results in a higher accumulation of calcite particles from the water, which further contributes to the erosion of the polyester surface.

**Figure 8:**
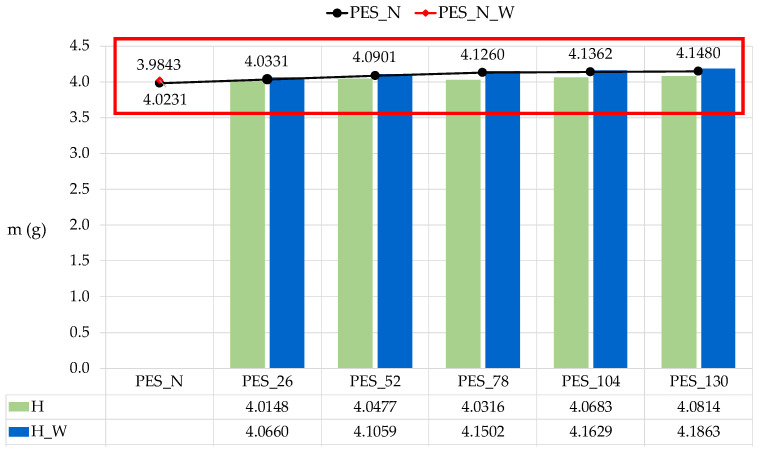
The changes in polyester fabric weight as a function of aging (H) and aging–washing (H_W) process.

The polyester fabrics were analyzed using gravimetric methods, as the standards for microplastics originating from textiles require monitoring the material alterations of the fabrics during the washing process [60,61,62]. Figure 8 illustrates the changes in weight of the polyester fabric (PES_N) resulting from both aging and the washing process.

Irradiation causes the photooxidative degradation of polyester, starting at the surface of the material, as oxygen diffuses slowly into the polymer structure. Figure 8 shows the weight of the aged polyester fabrics before and after 10 washing cycles. The weight of the untreated polyester fabric before and after washing is also shown. The gravimetric indicator of all washed samples shows the increase in mass compared to the untreated and aged samples. In contrast, the fragments created during aging are released during the washing process. The observed increase in mass could be due to the accumulation of deposits that result from washing with the standard ECE-A detergent in tap water at a temperature of 60 °C. The deposits on the surface of the polyester fabrics are visible on the SEM images (Figure 7b), although the standard detergent contains a builder. This is a non-soluble ion exchanger, an aluminosilicate (zeolite A), which is responsible for removing calcium and magnesium salts from the tap water. The effect of the ion exchanger was poor during the washing process, so that the calcite formed and deposited on the surface of the polyester fabric.

**Figure 9:**
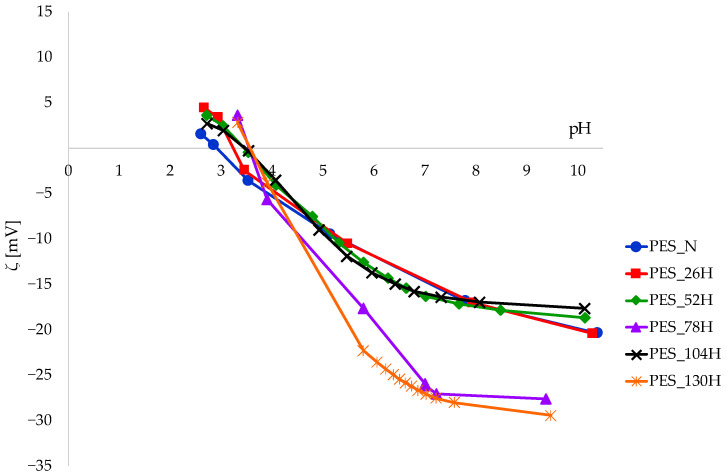
The zeta potential of polyester fabrics before and after aging as a function of pH 1 mmol/L KCl.

**Figure 10:**
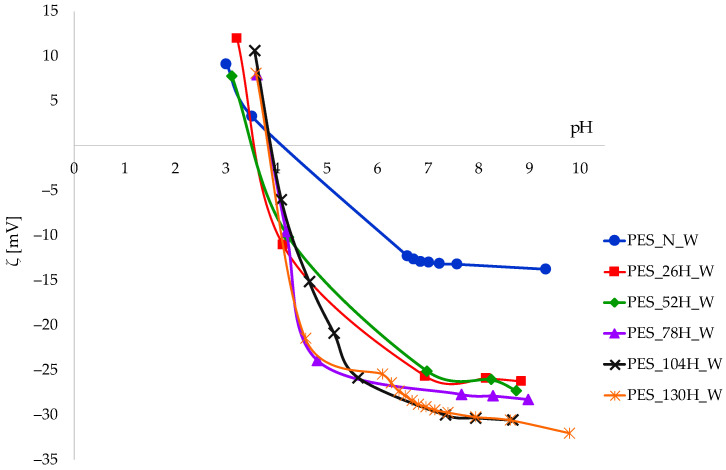
The zeta potential of polyester fabrics after aging and washing as a function of pH 1 mmol/L KCl.

The zeta potential was chosen as a parameter for monitoring the surface properties of polyester fabrics during the aging and washing process; see Figure 9 and Figure 10.

The negative zeta potential value rises as the pH of the electrolyte solution increases during measurement, which was to be expected based on the theory of electrokinetic potential. The zeta potential measurements for PES_N at a pH of 10.4 are approximately −20.41 mV. In contrast, the standard PES fabric, which possesses different structural characteristics in the alkaline range, exhibits a zeta potential of −69.0 mV [63]. Previous research has demonstrated that the standard polyester fabric in use contains a small fraction of surface preparations that impede the complete dissociation of active groups [64].

The zeta potential curves of aged samples (PES_26H, PES_52H and PES_104H) are similar to untreated polyester fabric (PES_N). The zeta potential values of samples aged 78 and 130 h are more negative compared to the untreated one. During the sun/rain cycle, the water washes away the part degraded by the sun and hydrolyses the surface of the polyester fabric, making it more accessible through the surface erosion of the layer, i.e., the hydrolysis of the ester bonds.

The isoelectric points (IEPs) of all samples examined fall within the pH range of 3 to 4. The previously noted effect of aging, specifically at 78 and 130 h, is also reflected in the IEP, which shows a shift towards a higher pH when compared to both untreated samples and those aged for different durations (26H, 52H, 104H).

In spite of the differences in IEPs and the zeta potential values for 78H and 130H, the aging process carried out according to the dry–wet protocol did not impact the regular surface behavior, as was the case with tensile properties. The images of the SEM samples are distinct from those of aged samples. Particle deposits are accumulated on the surface of all washed samples (untreated and aged) over 10 cycles. The impact of the washing process on the zeta potential of both untreated and aged samples is illustrated by the curves presented in Figure 10.

The surface properties of untreated polyester fabric remain largely unaffected by the washing process, notwithstanding the presence of particle deposits detected by SEM.

The curves of the washed aged samples are characterized by a greater degree of clustering compared to curves shown in Figure 9. The zeta potential of all aged fabrics after washing is more negative when compared to aged ones. The washed samples aged for 78 h (78H_W) and 130 h (130H_W) exhibited the smallest degree of change.

According to the zeta potential values, the changes on the surface of the aged washed samples could be attributed to detergency and the further alkali hydrolysis of the aged polyester fabrics at 60 °C, although particle deposits were detected in the SEM. It can be concluded that the influence of the washing process should be taken into account, due to the smaller differences in surface charge compared to the surface charge of the aged samples.

The specific surface area (SSA) of the polyester samples, including those with the shortest and longest aging times, as well as the initial sample before and after washing, was determined based on the quantity of nitrogen adsorbed using the BET method; see Table 4. The findings indicate that the standard polyester fabric has a specific surface area of 5.6069 m^2^/g, which diminishes as aging and washing increase, ultimately reaching a value of 0.3438 m^2^/g. Washing in an alkaline bath reduces the specific surface area of the samples. Larger differences are observed during aging, which can be attributed to the aging conditions. The hydrolysis of the polyester surface is caused by a combination of sun/rain as the water washes away the part degraded by the sun. This surface modification improves the ability to interact with detergent components, which is also an indicator of reduced nitrogen adsorption. Minor differences in SSA between aged and washed samples (PES_N_W, PES_26H_W, and PES_130H_W) can be attributed to the presence of calcite from the wash bath. The BET analysis correlates with the SEM and zeta potential results.

### 3.2. Characterization of Wastewater

Considering the fibrillation of aged samples and the hydrodynamics of the washing process, which are enhanced by the action of the detergent and the migration of fibers from the polymer structure in the washing process, the contamination of the wastewater is possible. In order to analyze the presence of protruding fibers released from the polyester samples during the washing process, the wastewater collected in 10 washing cycles was examined microscopically and analyzed using the polyethersulfone filter. Table 5 presents an analysis of a wastewater sample and a filter, examined using an optical microscope.

Table 5 shows some fibrillar and non-fibrillar forms in the collected wastewater samples. The detached fiber fragments from the polyester samples were isolated. According to this interpretation, the release does not have a direct correlation with the duration of irradiation. Therefore, the other parameters of the physico-chemical analysis (pH, conductivity, TSS, TS, TDS, turbidity and COD) were determined, as presented in Table 6.

Table 6 shows the physico-chemical parameters of the wastewater from the washing process. The results show that most of the parameters of the analyzed wash wastewater from aged polyester fabrics are changed compared to the original polyester fabric wastewater sample (PES_N). The parameters of turbidity and total suspended solids (TSS) are linked, whereby the degree of turbidity can be seen as an indicator of changes in the concentration of suspended solids in the wastewater.

The smallest changes can be seen in the TSS parameter, while the largest changes occur in turbidity and COD. The electrical conductivity of the wastewater from the washing process of untreated PES fabric is the highest, which can be attributed to the release of compounds from the surface of the fabric into the wastewater. The highest rate of increase was found for the turbidity parameter. The turbidity increase is linked to the aging hours caused by the alkali hydrolysis of polyester, showing the most substantial change after 130 h (PES_130H_W). The observed correlation can be linked to the release of generated fragments, which are particles originating from the surface and are hydrolyzed in the alkaline conditions of the wash bath, characterized by a pH of 8.3 in the 1.25 g/L detergent solution. An additional factor to consider could be the composition of the detergent, given that the total solids (TS) measurement in wastewater from older samples is greater than that of the original sample. The solids can originate both from detergent components (aluminosilicates) and from fragments released by the aging of the PES fabric. The changes in TS and TDS values of wastewater did not increase with aging time, which may be related to a more complex degradation process that not only increases with time but reflects the nuanced degradation patterns of the parent material under washing conditions. Significant changes were identified in the COD parameter, showing markedly higher values in wastewater samples that had been aged and washed for an extended duration. The wastewater exhibits a four- to seven-fold rise in COD, indicating that aging is linked to the presence of chemical contaminants. This situation can be attributed to the compounds that are released from the surfaces of the aged samples, along with the presence of soluble detergent components.

## 4. Conclusions

The standard polyester fabric underwent a controlled artificial aging process lasting between 26 and 130 h, along with exposure to sun and rain cycles, as well as an modified washing process. This approach aimed to investigate the characteristics of aged and washed polyester fabrics, as well as the pollution levels in the washing wastewater.

The results of tensile strength, structural units, and the surface (SEM images, zeta potential) and gravimetric indicators proved that irradiation causes the photooxidative degradation of the polyester, which starts at the surface of the material due to the slow diffusion of oxygen into the polymer structure. The tensile strength of aged polyester fabrics decreases in comparison to untreated samples, with a greater reduction observed as the duration of irradiation increases. The effects of modified washing process were also confirmed by the tensile and surface properties as well as the structural units of the aged polyester fabrics. The rupture of fibrils within the yarn structure led to the formation of loops around adjacent threads and the reinforcement of crossing points within the fabric. As a result, the fabric became stronger. The washing process led to a deposition of calcite particles on the surface of the polyester fabrics, which was verified by SEM images and the zeta potential. The chemical oxygen demand (COD) and turbidity indicated impurities in the wastewater originating from detergent components and fragments from aged polyester fabrics.

## Figures and Tables

**Table 1:** Sample labels and description.

Samples	Description of Polyester Fabric
PES_N	Untreated fabric
PES_26H	Fabric aged 26 h
PES_52H	Fabric aged 52 h
PES_78H	Fabric aged 78 h
PES_104H	Fabric aged 104 h
PES_130H	Fabric aged 130 h
PES_N_W	Untreated fabric washed 10 times
PES_26H_W	Fabric aged 26 h and washed 10 times
PES_52H_W	Fabric aged 52 h and washed 10 times
PES_78H_W	Fabric aged 78 h and washed 10 times
PES_104H_W	Fabric aged 104 h and washed 10 times
PES_130H_W	Fabric aged 130 h and washed 10 times

**Table 2:** Digital image of polyester fabrics before and after artificial aging and washing.

Untreated	40×	Washed	40×
PES_N	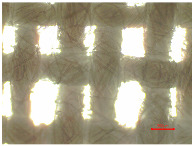	PES_N_W	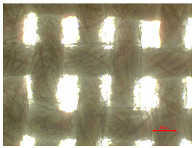
**Aged**	**40×**	**Aged and washed**	**40×**
PES_26H	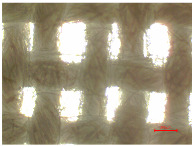	PES_26H_W	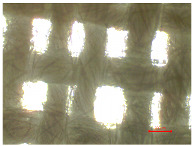
PES_52H	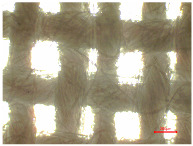	PES_52H_W	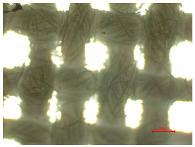
PES_78H	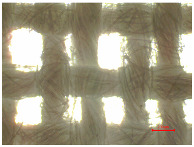	PES_78H_W	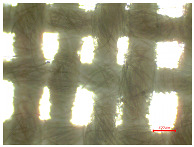
PES_104H	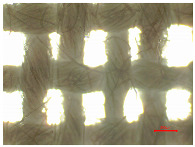	PES_104H_W	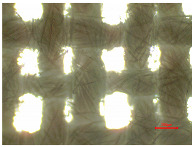
PES_130H	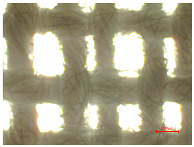	PES_130H_W	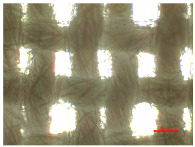

**Table 3:** Breaking force (F_b_) and elongation (ε_b_) of polyester fabric before and after artificial aging and washing.

Samples	F_b_ (MPa)	ε_b_ (%)	Samples	F_b_ (MPa)	ε_b_ (%)
PES_N	58.4571	19.33	PES_N_W	58.6111	22.95
PES_26H	51.6111	20.39	PES_26H_W	54.9189	22.75
PES_52H	50.2778	20.49	PES_52H_W	54.0541	21.10
PES_78H	47.4595	21.32	PES_78H_W	49.0000	21.79
PES_104H	42.7568	19.35	PES_104H_W	46.8947	20.75
PES_130H	40.5946	19.27	PES_130H_W	42.5789	21.05

**Table 4:** BET analysis of polyester fabrics.

Samples	SSA (m^2^/g)	Qm (mmol/g)	R^2^	Langmuir Surface Area (m^2^/g)
PES_N	5.6069	0.05746	0.9987739	9.3266
PES_26H	1.5187	0.01557	0.9976390	2.5940
PES_130H	0.4665	0.00478	0.9922991	1.0586
PES_N_W	0.3438	0.00352	0.9982508	0.5970
PES_26H_W	0.4463	0.00457	0.9959854	0.9176
PES_130H_W	0.4206	0.00431	0.9977252	0.8479

**Table 5:** A microscopic analysis of the effluent from wastewater and the filter cake.

Samples	Wastewater (40×)	Filter (40×)
PES_N_W	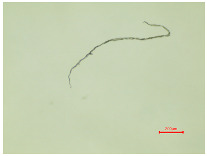	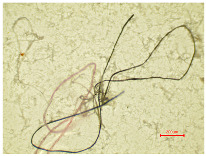
PES_26H_W	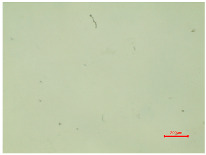	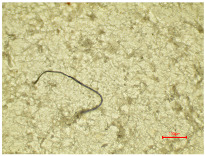
PES_52H_W	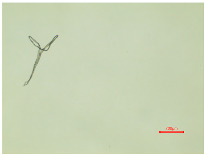	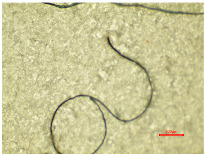
PES_78H_W	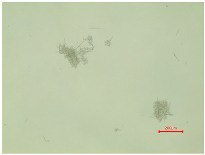	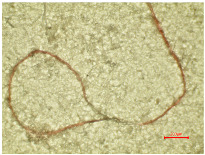
PES_104H_W	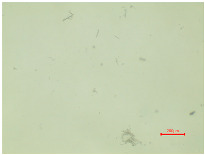	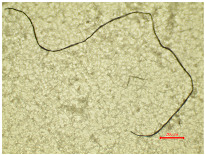
PES_130H_W	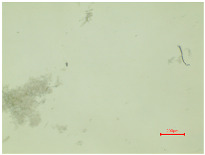	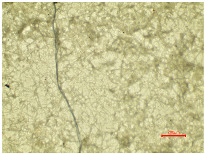

**Table 6:** Physico-chemical characteristics of wastewater.

Samples	PES_N	PES_26H_W	PES_52H_W	PES_78H_W	PES_104H_W	PES_130H_W
TDS (mg/L)	52.00	69.12	59.75	69.11	63.09	69.13
TS (mg/L)	88.12	93.23	98.84	94.42	94.41	99.11
TSS (mg/L)	54.14	55.12	50.00	55.34	52.16	54.15
pH	8.41	8.58	8.37	8.37	8.44	8.51
κ (µS/cm)	625.1	621.9	625.1	521.3	553.4	581.5
T (NTU)	42	57	59	60	62	90
COD (mg O_2_/L)	150	633	584	1014	1054	1164

## Data Availability

Data are contained within the article.
